# Active macropinocytosis induction by stimulation of epidermal growth factor receptor and oncogenic Ras expression potentiates cellular uptake efficacy of exosomes

**DOI:** 10.1038/srep10300

**Published:** 2015-06-03

**Authors:** Ikuhiko Nakase, Nahoko Bailey Kobayashi, Tomoka Takatani-Nakase, Tetsuhiko Yoshida

**Affiliations:** 1 Nanoscience and Nanotechnology Research Center, Research Organization for the 21st Century, Osaka Prefecture University, Naka-ku, Sakai, Osaka 599-8570, Japan; 2 Keio Advanced Research Centers (KARC), Keio University, Tsukuba, Ibaraki 300-2611, Japan; 3 Institute for Advanced Sciences, Toagosei Co., Ltd., Tsukuba, Ibaraki 300-2611, Japan; 4 Department of Pharmaceutics, School of Pharmacy and Pharmaceutical Sciences, Mukogawa Women’s University, 11-68, Koshien Kyuban-cho, Nishinomiya, Hyogo 663-8179, Japan

## Abstract

Exosomes are approximately 100-nm vesicles that consist of a lipid bilayer of cellular membranes secreted in large quantities from various types of normal and disease-related cells. Endocytosis has been reported as a major pathway for the cellular uptake of exosomes; however, the detailed mechanisms of their cellular uptake are still unknown. Here, we demonstrate the active induction of macropinocytosis (accompanied by actin reorganisation, ruffling of plasma membrane, and engulfment of large volumes of extracellular fluid) by stimulation of cancer-related receptors and show that the epidermal growth factor (EGF) receptor significantly enhances the cellular uptake of exosomes. We also demonstrate that oncogenic K-Ras-expressing MIA PaCa-2 cells exhibit intensive macropinocytosis that actively transports extracellular exosomes into the cells compared with wild-type K-Ras-expressing BxPC-3 cells. Furthermore, encapsulation of the ribosome-inactivating protein saporin with EGF in exosomes using our simple electroporation method produces superior cytotoxicity via the enhanced cellular uptake of exosomes. Our findings contribute to the biological, pharmaceutical, and medical research fields in terms of understanding the macropinocytosis-mediated cellular uptake of exosomes with applications for exosomal delivery systems.

In cell-to-cell communication, exosomes have received significant attention as an important carrier of bioactive molecules, including membrane receptors, proteins, and microRNA; they are capable of manipulating cell functions not only for the maintenance of biological homeostasis but also in relation to diseases^1^. Exosomes are ~100-nm vesicles that consist of a lipid bilayer of cellular membranes generated from multivesicular bodies in cells^1^, and they are secreted in large quantities (there are approximately 3,000,000 exosomes/μl in the blood^2^) from various types of normal and disease-related cells. Exosomes can contain lipids (sphingomyelin, cholesterol, ceramide), membrane proteins (Alix, TSG 101), tetraspanins (CD63, CD37, CD53, CD81, CD82), heat-shock proteins (Hsp84/90, Hsc70), antigens (MHC I and MHC II), and enzymes (phosphate isomerase, peroxiredoxin, aldehyde reductase, fatty acid synthase)^1^,^3^. In a current immunotherapy trial, the possibility of exosome-based cancer vaccines (e.g., exosomes bearing tumour antigens to recognise and destroy cancer cells) has been exploited^4^,^5^,^6^,^7^,^8^. Oncogenic microRNAs can also be delivered by exosomes during cancer invasion and metastasis^9^,^10^,^11^. In addition, the encapsulation of specific types of microRNAs in exosomes, which are secreted from disease-related cells (especially from tumours), has also been reported [e.g., miR-1246 (oesophageal squamous cell carcinoma) and miR-1229 (colon cancer)]. The detection of exosomal microRNA from human bodily fluids, including blood, breast milk, saliva, urine, placenta, amniotic fluid, and nasal fluid, is being intensively investigated for next-generation diagnosis technology for detecting silent human diseases with a low patient burden^3^,^12^,^13^. In addition, exosomes have been studied as delivery carriers of genes for cellular regulation and therapeutics^1^,^14^,^15^,^16^,^17^. For example, exosomes have been applied to knockdown BACE1 as a therapeutic target of Alzheimer’s disease^18^. Exosomes are highly anticipated to be next-generation therapeutic carriers with certain pharmaceutical advantages, including non-immunogenicity, constitutive secretion from cells, very low cytotoxicity, original and artificial encapsulation of bioactive molecules (especially microRNAs), and the protein engineering of the exosomal membrane^19^. However, exosome-mediated delivery systems should be further developed, especially in terms of their low efficiency of cellular uptake with competition among a markedly high number of pre-existing natural exosomes in human fluids, as described above.

Endocytosis has been reported to be a major pathway for the cellular uptake of exosomes, and exosomal membrane proteins (e.g., CD9, CD81) have been reported to be ligands for their endocytosis pathways^20^,^21^,^22^,^23^. However, the detailed mechanisms of their cellular uptake are still unknown. Further elucidation of the complicated mechanisms involved is urgently required to understand exosome-based cell-to-cell communications from the standpoint of cellular uptake control and for the development of intracellular delivery systems of therapeutic and diagnostic molecules based on exosomal internalisation mechanisms.

In our novel findings related to exosome cellular uptake mechanisms, we here demonstrate the active induction of macropinocytosis (accompanied by actin reorganisation, ruffling of the plasma membrane, and engulfment of large volumes of extracellular fluid^24^,^25^) by stimulation of cancer-related receptors, including the epidermal growth factor receptor (EGFR), which significantly enhances the cellular uptake of exosomes (Fig. 1). Especially after EGFR stimulation by treatment with the receptor ligand epidermal growth factor (EGF), the cellular uptake of exosomes was enhanced approximately 27-fold in A431 human epidermoid carcinoma cells that express high levels of EGFR on the plasma membrane (with expression of over 10^6^ receptors/cell)^26^. MIA PaCa-2 cells (human pancreatic adenocarcinoma cells), which are homozygous for the K-Ras^G12C^ allele, have been reported to exhibit high levels of macropinocytosis for the transport of extracellular proteins into the cells as an important route of nutrient uptake compared with BxPC-3 cells, which express wild-type K-Ras^27^,^28^,^29^. Our experimental results showed a highly superior cellular uptake of exosomes by MIA PaCa-2 cells compared with that of BxPC-3 cells, suggesting the possible relevance of cell-to-cell communication based on exosomes in oncogenic Ras-mediated macropinocytosis and malignant progression. The ribosome-inactivating protein saporin-encapsulated exosomes also showed enhanced cytotoxicity by the addition of EGF for the induction of macropinocytosis. In addition, we here demonstrate that the electroporation of exosomes for the artificial encapsulation of EGF significantly enhanced their cellular uptake, and the encapsulation of saporin with EGF in exosomes using our simple electroporation method resulted in superior induction of cytotoxicity compared with that of saporin-encapsulated exosomes without co-encapsulation of EGF.

## Results

### Activation of the macropinocytosis pathway by EGFR stimulation enhances the cellular uptake of exosomes

Endocytosis has been shown to be a major pathway for the cellular uptake of exosomes, as described above^20^,^21^,^22^,^23^. However, the endocytosis pathway has a cargo size limitation (~120 nm) for cellular uptake^30^. Exosomes are approximately 100-nm vesicles, and the endocytosis pathway exhibits low efficacy for the cellular uptake of exosomes because of the size limitation. The macropinocytosis pathway involves the engulfment of large volumes of extracellular fluid, and this pathway achieves cellular uptake of large cargo sizes (~1 μm)^24^,^25^,^30^. In this study, we investigated the effects of macropinocytosis induction on the cellular uptake efficiency of exosomes. EGFR, which is a representative tyrosine kinase receptor, regulates signal transduction pathways for proliferation, motility, and survival^31^,^32^,^33^. EGFR expression has been observed in a variety of human tumours, such as those in the lung, head and neck, pancreas, ovary, and glioma, and high expression of the receptor has been shown to be associated with poor prognosis in patients with tumours^34^. EGFR stimulation by treatment with the receptor ligands, including EGF, induces signal transduction pathways for macropinocytotic cellular uptake via the activation of Rac, a member of the Rho family of small GTPases, which leads to actin cytoskeletal organisation containing lamellipodial extensions^35^,^36^. The effects of EGFR stimulation on the cellular uptake of exosomes were examined first and are shown in Fig. 2. A431 human epidermoid carcinoma cells highly express EGFR on the plasma membrane^26^, and EGF effectively induces receptor activation (Supplementary Fig. 2a) and signalling for macropinocytotic cellular uptake. Lamellipodia formation and membrane ruffling for macropinocytotic cellular uptake is induced by EGF treatment in A431 cells (Supplementary Fig. 2b-d). In this research, green fluorescent protein (GFP)-fused CD63-expressing exosomes (CD63-GFP-exosomes) were used for the detection of their cellular uptake. Tetraspanin CD63 protein is a marker protein of exosomal membranes^1^, and GFP-fused CD63 stably expressing HeLa cells (CD63-GFP-HeLa) were prepared (Supplementary Fig. 1a). The CD63-GFP exosomes secreted from CD63-GFP HeLa cells were isolated as described in the Methods section. Isolated exosomes were observed using transmission electron microscopy (TEM; Supplementary Fig. 1b), and the expression of the exosomal marker protein CD63 in isolated exosomes was detected using western blot analysis (Supplementary Fig. 1c).

Figure 2a shows the confocal microscopic results of A431 cells treated with CD63-GFP-exosomes (20 μg/ml) in the presence or absence of EGF (500 nM) for 24 h at 37 °C in serum-free cell culture medium. In the absence of EGF, internalised CD63-GFP exosomes were difficult to observe because of the very low cellular uptake efficacy of the exosomes in this experimental condition (Fig. 2a). Negatively charged exosomal membranes^37^,^38^,^39^ are thought to repel negatively charged cellular membranes, resulting in a low cellular uptake efficiency of these exosomes. The zeta potential of isolated exosomes in this experiment was −11.6 mV, as analysed using a zeta potential analyser, which was a value similar to previously reported results^37^,^38^,^39^. On the other hand, the addition of the EGF significantly affected the cellular uptake efficiency of the exosomes, and increased fluorescent signals of CD63-GFP-exosomes taken up by the cells were observed using a confocal microscope (Fig. 2a). Under the same experimental conditions as with the confocal microscopy observations, the fluorescence intensity of the CD63-GFP-exosomes inside the cells was detected using a flow cytometer as described in Methods section (Fig. 2b). The analysis showed enhanced cellular uptake efficiency of the exosomes through the addition of EGF (e.g., approximately 27-fold enhanced cellular uptake by the addition of 500 nM EGF; Fig. 2b). In addition, an increase in the cellular uptake efficiency of the exosomes (approximately a 5-fold increase) by the addition of EGF was observed even under in the presence of serum [10% foetal bovine serum (FBS), which contains a high amount of pre-existing bovine serum exosomes] (Fig. 2b). CD63-GFP-exosomes that were isolated by ultracentrifugation as described in the Methods section were also tested, and similar results showing enhanced cellular uptake efficiency of the exosomes were observed under the same experimental conditions as described for Fig. 2b (data not shown). The continuous addition of EGF (500 nM/day) also significantly enhanced the cellular uptake of exosomes (approximately 40-fold) compared with the absence of EGF (Supplementary Fig. 3). Additionally, increased EGFR expression by transfection of the EGFR gene increased the internalisation of CD63-GFP-exosomes (20 μg/ml) in the presence of EGF (500 nM; Supplementary Fig. 4).

Dextran (70 kDa) is a marker molecule for the macropinocytosis pathway^40^,^41^. Supplementary Fig. 2c shows the confocal microscopic results of A431 cells treated with Texas Red-labelled dextran (70 kDa, 0.5 mg/ml) for 24 h at 37 °C in the presence or absence of EGF (500 nM). In the absence of EGF, the fluorescent signals of the Texas Red-labelled dextran taken up by the cells were low (Supplementary Fig. 2c). On the other hand, the addition of EGF increased the cellular uptake of the Texas Red-labelled dextran (Supplementary Fig. 2c). Under the same experimental conditions, the cellular uptake of FITC-labelled dextran (70 kDa, 0.5 mg/ml) was analysed using a flow cytometer, and the addition of the EGF enhanced the internalisation of the dextran by cells based on the EGF concentration (Supplementary Fig. 2d). When A431 cells were treated with CD63-GFP-exosomes (20 μg/ml) and Texas Red-labelled dextran (70 kDa, 0.5 mg/ml) in the presence of EGF (500 nM), fluorescent exosome signals and dextran taken up by the cells were highly colocalised inside the cells (Fig. 2c), suggesting that the cellular uptake of exosomes in this experimental condition occurs through the macropinocytosis pathway. In addition, the internalisation efficacy of exosomes by the cells in the presence of EGF was significantly reduced by treatment with the macropinocytosis inhibitor 5-(*N*-ethyl-*N*-isopropyl)amirolide (EIPA)^24^,^42^ (Fig. 2d).

### Macropinocytosis induction via CXCR4 increases the cellular uptake of exosomes

CXCR4 is a chemokine receptor (C-X-C chemokine receptor type 4), and an increased expression of this receptor has been related to cancer progression^43^,^44^. Stromal cell-derived factor 1α (SDF-1α) is a natural CXCR4 ligand, and the stimulation of CXCR4 by treatment with SDF-1α induces CXCR4 endocytosis and actin polymerisation^45^,^46^, leading to macropinocytotic cellular uptake^47^. The effect of CXCR4 stimulation by SDF-1α on the cellular uptake of exosomes was also examined. HeLa cells were treated with CD63-GFP-exosomes (20 μg/ml) for 24 h, followed by flow cytometer analysis. The results showed that the addition of SDF-1α increased the internalisation of exosomes by the cells (approximately 2.3-fold by co-treatment with SDF-1α (200 nM) with the exosomes) even in 10% FBS-containing cell culture medium, suggesting that CXCR4-dependent macropinocytosis also increases the cellular uptake of exosomes.

### Enhanced cellular uptake of exosomes via the macropinocytosis pathway by oncogenic Ras

The expression of the oncogenic Ras protein in tumour cells has been shown to induce macropinocytosis^48^,^49^,^50^. Commisso *et al.* recently reported that Ras-transformed tumour cells transport extracellular proteins through the macropinocytosis pathway^29^. The macropinocytosis pathway was shown to be an important route for the cellular uptake of nutrients in tumours, and the growth of the Ras-transformed pancreatic tumour xenografts was inhibited by treatment with the macropinocytosis inhibitor EIPA^29^. In this research, we examined the cellular uptake efficacy of exosomes by Ras-transformed tumour cells (Fig. 3). MIA PaCa-2 human pancreatic adenocarcinoma cells are homozygous for the K-Ras^G12C^ allele, and the cells highly induce macropinocytosis to transport extracellular proteins into the cells as an important route of nutrient uptake^27^,^28^,^29^. In Fig. 3, we show the cellular uptake efficiency of exosomes by MIA PaCa-2 cells compared with that of BxPC-3 cells, which express wild-type K-Ras, to assess the effects of oncogenic Ras expression and macropinocytosis in the cellular uptake of exosomes. Figure 3a shows the relative cellular uptake of CD63-GFP-exosomes (20 μg/ml) by MIA PaCa-2 or BxPC-3 cells in the presence or absence of EGF (500 nM) for 24 h at 37 °C analysed using flow cytometer. Both in the presence and absence of EGF stimulation, the efficiency of the cellular uptake of exosomes in the MIA PaCa-2 cells was superior (approximately 14 fold in the absence of EGF) to that of the BxPC-3 cells (Fig. 3a). In this experimental condition, internalisation of FITC-labelled dextran, which is a marker of macropinocytosis, by MIA PaCa-2 cells was shown to be higher than in BxPC-3 cells (Fig. 3b). On the other hand, internalisation of FITC-labelled transferrin, which is a marker of clathrin-mediated endocytosis, by MIA PaCa-2 cells was shown to be substantially lower than that of BxPC-3 cells (Fig. 3c). Colocalisation of internalised exosomes and Texas Red-labelled dextran inside MIA PaCa-2 cells was observed using a confocal microscope (Fig. 3d). These results suggest that macropinocytosis increased by oncogenic Ras enhances the cellular uptake efficacy of exosomes similar to the uptake of nutrients by tumour cells.

### Macropinocytosis induction enhances cytotoxicity of saporin-encapsulated exosomes

CD63-GFP-exosomes secreted from HeLa cells did not induce cytotoxicity in A431 cells even under the experimental condition of serum-free medium with the addition of EGF (Fig. 4a). We next examined the delivery of bioactive molecules by encapsulation in exosomes. Saporin (approximately 30 kDa) is a ribosome-inactivating protein that inhibits protein synthesis and the growth of targeted cells^51^. Encapsulation of saporin in exosomes was conducted by electroporation. As described in the Methods section, the electroporation method was optimized for poring pulse (50, 100, 200, 300 V), resulting in encapsulation of FITC-labeled saporin (45, 65, 120, 105 ng/ml) in 20 μg/ml of exosomes, respectively. TEM analysis showed almost no morphological change after the electroporation condition (poring pulse, 200 V) (Supplementary Fig. 5a). We also assessed aggregation of the proteins after electroporation as pointed out by Kooijmans *et al.*^52^ (Supplementary Fig. 5b). Kooijmans *et al.* reported that extensive aggregation of siRNAs by electroporation (400 V) analysed using confocal microscope^52^. We found that high voltage (300 V) electroporation showed aggregation of FITC-saporin-exosomes (Supplementary Fig. 5b). In the condition of 200 V electroporation, aggregation of FITC-saporin-exosomes was significantly improved (Supplementary Fig. 5b). The aggregation might reduce encapsulation efficacy of FITC-saporin in exosomes in the electroporation condition of 300 V. Therefore, we used an experimental poring pulse of 200 V in the electroporation steps. A431 cells were treated with saporin-encapsulated exosomes (4 μg/ml of total saporin-encapsulated exosomes) or saporin (7 μg/ml of saporin) in 10% FBS-containing cell culture medium for 48 h at 37 °C, prior to a WST-1 assay (Fig. 4b). In the treatment of saporin-encapsulated exosomes, the addition of EGF (500 nM) significantly enhanced cytotoxicity (Fig. 4b), suggesting that the induction of macropinocytosis by EGF stimulation increases the cellular uptake of saporin-encapsulated exosomes and saporin bioactivity inside cells. In contrast, a high concentration of saporin only slightly increased cytotoxicity by EGF stimulation in this experimental condition (Fig. 4b).

### EGF encapsulation enhances the cellular uptake efficacy of exosomes

Next, we examined the effects of EGF encapsulation in exosomes on the cellular uptake efficacy of the exosomes without modification of EGF on exosomal membranes (Fig. 5). The localised delivery of EGF for tissue and cell repair is a very important technique, as growth factor therapies activate and regulate a variety of cellular functions^53^,^54^. Marquez *et al.* recently reported the potential usage of liposomes to carry EGF to enhance the bone-healing process in injured rat alveoli, and EGF-encapsulated liposomes produced a more rapid recovery of the wound^55^. EGF has been shown to recycle by exocytosis after cellular uptake via endocytosis^56^. Therefore, after the cellular uptake of EGF-encapsulated liposomes, the release of EGF from liposomes in endosomes possibly induces EGF recycling, and secreted EGF from the cells by exocytosis stimulates EGFR expression at cellular plasma membranes. We also tested our hypothesis that encapsulated EGF in exosomes releases EGF from endosomes after cellular uptake, and recycled EGF stimulates the EGFR, leading to an enhanced macropinocytosis pathway and exosome uptake by cells. Encapsulation of EGF in exosomes was achieved by electroporation as described in the Methods section, and internalisation of the exosomes by A431 cells was assessed. In addition, transferrin (clathrin-mediated endocytosis)-encapsulated exosomes was also prepared by electroporation similar to that of EGF. Figure 5a shows the confocal microscopic images of A431 cells treated with EGF- or transferrin-encapsulated CD63-GFP-exosomes (20 μg/ml) for 24 h at 37 °C. Based on the results, the highest fluorescent intensity of internalised EGF-encapsulated CD63-GFP-exosomes inside the cells was observed compared with that of CD63-GFP-exosomes without encapsulation as a control and with encapsulation of transferrin (Fig. 5a). The relative cellular uptake of each exosome was also assessed using a flow cytometer, and EGF encapsulation enhanced the cellular uptake of the exosomes (approximately 8 fold of control; Fig. 5b). On the other hand, transferrin encapsulation slightly increased exosome internalisation (Fig. 5b). These results suggest that EGF encapsulation significantly affects the cellular uptake of exosomes, and this simple technique is considered very useful for enhancing exosome internalisation by targeted cells.

The results of exosome-mediated saporin delivery using the method of EGF encapsulation are also shown in Fig. 6. Similar to Fig. 4b, saporin-encapsulated CD63-GFP-exosomes were prepared in the presence or absence of EGF by the electroporation method as described in the Methods section. Fig. 6a shows the microscopic observation of A431 cells treated with saporin- or both saporin- and EGF-encapsulated exosomes (0.4, 4, 20 μg/ml) for 72 h at 37 °C in 10% FBS-containing cell culture medium. Surprisingly, a low concentration (0.4 μg/ml) of saporin-EGF-exosomes prevented A431 cellular proliferation, and 4 μg/ml of saporin-EGF-exosomes induced significant levels of cell death (Fig. 6a). On the other hand, 0.4 and 4 μg/ml of saporin-exosomes without EGF encapsulation did not induce morphological changes in the A431 cells, and a high concentration (20 μg/ml) of saporin-exosomes inhibited A431 cellular proliferation (Fig. 6a). A WST-1 assay for the detection of cell viability was also conducted using the same experimental conditions as in Fig. 6a, and co-encapsulation of EGF in exosomes effectively enhanced the cytotoxicity of encapsulated saporin to the A431 cells even at a low exosome concentration (0.4 μg/ml; Fig. 6b). The results support the findings of enhanced cellular uptake of exosomes by encapsulation of EGF in Fig. 5, and this simple technique is considered a very useful method for the delivery of biofunctional proteins into cells to successfully attain the bioactivity of encapsulated therapeutic molecules in targeted cells.

## Discussion

**Figure 1:**
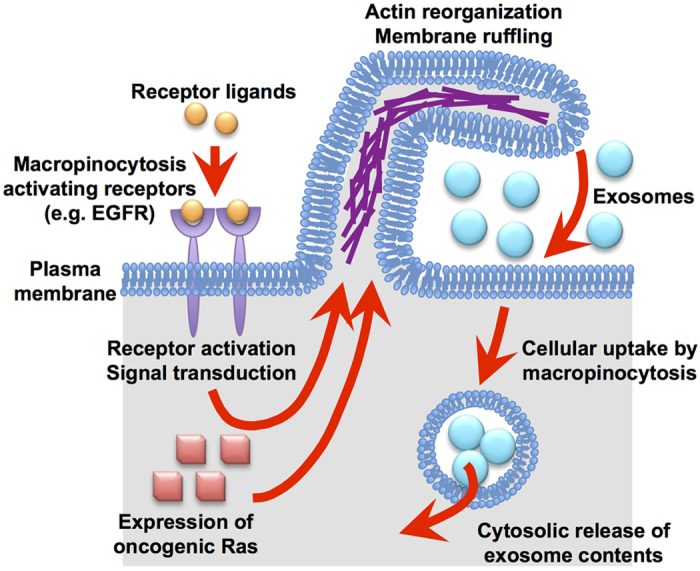
Schematic representation of enhanced cellular uptake of exosomes by activation of the macropinocytosis pathway.

**Figure 2:**
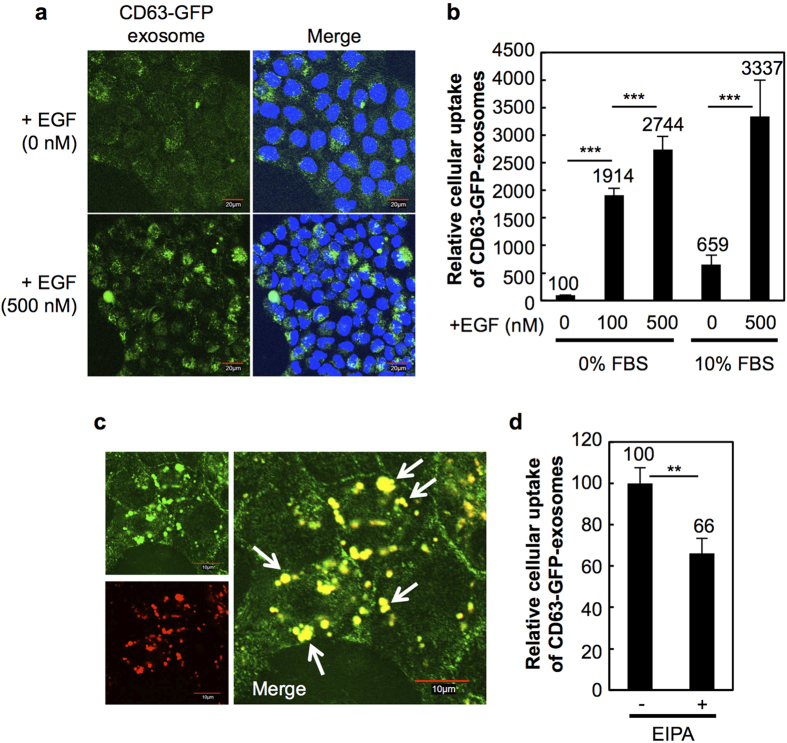
Stimulation of EGFR by treatment with EGF enhances cellular uptake of exosomes via the macropinocytosis pathway. (**a**) Confocal microscopic observation of A431 cells treated with CD63-GFP-exosomes (20 μg/ml) in the presence or absence of EGF (500 nM) for 24 h at 37 °C. Green signals, CD63-GFP-exosomes; blue signals, Hoechst 33342 for nuclear staining. Scale bar, 20 μm. (**b**) Relative cellular uptake of CD63-GFP-exosomes in the same experimental condition of (**a**) analysed using a flow cytometer. (**c**) Internalisation of CD63-exosomes (20 μg/ml) and Texas Red-dextran (70 kDa, macropinocytosis marker) in the presence of EGF (500 nM) by A431 cells analysed using a confocal microscopy after treatment for 24 h at 37 °C. Arrows show representative colocalisation of exosomes and dextran inside cells. Scale bar, 10 μm. (**d**) Effects of the macropinocytosis inhibitor, EIPA (100 nM), on the cellular uptake of CD63-GFP-exosomes (20 μg/ml) with EGF (100 nM) for 3 h at 37 °C, analysed using a flow cytometer. (**b,d**) The data are the averages (±SD) of three experiments. ^**^p < 0.01, ^***^p < 0.001.

Cellular uptake mechanisms of exosomes have been studied particularly in the endocytosis pathway, as noted in the Introduction section^20^,^21^,^22^,^23^, and certain membrane exosome proteins, including milk fat globule (MFG)-E8/lactadherin, CD11a, CD54, CD9, and CD81^20^, have been shown to possibly participate in the cellular uptake of exosomes as ligand proteins. Ligand-receptor interactions on the recipient cell surface are considered trigger points for their cellular uptake. The expression levels of the ligand proteins on the exosome membrane and receptors on the recipient cell surface possibly affect the efficacies of cell membrane accumulation of the exosomes. However, even though ligand-receptor interaction leads to binding and accumulation of exosomes on the recipient cell surface, whether receptor-mediated endocytosis is induced by receptor activation is an important point that should be elucidated to understand the cellular uptake mechanisms for exosomes. On the other hand, we here demonstrated active cellular uptake of exosomes through the macropinocytosis pathway, which is induced by stimulation of macropinocytosis-related receptors and oncogenic Ras protein (Fig. 1) that highly contribute to tumour progression. The physiologic environment, including the concentration of extracellular proteins, positively affects cellular signalling through the receptors, and expression levels of the receptors determine cellular responses, including cellular uptake. As shown in Fig. 2, A431 cells highly express EGFR on the plasma membrane, and stimulation of the receptor by treatment with the receptor ligand EGF significantly enhanced exosome internalisation by the cells (Fig. 2a, b). The expression level of macropinocytosis-related receptors and the concentration of their receptor ligands are important factors for the cellular uptake of exosomes, which might regulate cellular responses by exosomal content after their cellular uptake. Thus, consideration of not only exosomal membrane proteins that participate in their cellular uptake but also activation of macropinocytosis-related receptors by their receptor ligands, which exist in human fluids, including blood, are very important for the elucidation of cellular uptake mechanisms and cell-to-cell communications.

Activation of tyrosine kinase receptors, such as EGFR and platelet-derived growth factor receptor, intensely increases actin polymerisation in cell peripheral membrane regions, leading to actin-mediated membrane ruffling and macropinosome formation^24^,^25^. Macropinocytosis can uptake a large volume (more than 1 μm in size) of extracellular fluid molecules into cells^30^. On the other hand, endocytosis, including clathrin-mediated endocytosis and caveolin-mediated endocytosis, has a size limitation of approximately ~100 nm for their cellular uptake, which is regulated by membrane curvature and self-assembly of protein scaffolds including clathrin coats^25^,^30^. Exosomes have been shown to be approximately ~100 nm in size^37^,^38^,^39^, leading to a low efficiency of their cellular uptake via endocytosis. Exosomal membranes are also negatively charged^37^,^38^,^39^, leading to hindered accumulation on negatively charged cell membranes. Considering these exosome characteristics, macropinocytosis is an effective pathway for the efficient cellular uptake of exosomes, which might relate to cell-to-cell communications. Even in the presence of serum, in which many bovine-derived exosomes exist and might compete with the cellular uptake of CD63-GFP-exosomes, stimulation of EGFR with the receptor ligand EGF significantly enhanced internalisation of the CD63-GFP-exosomes by A431 cells (Fig. 2b), suggesting the possibility of macropinocytosis-mediated control of their cellular uptake *in vivo*, although further detailed studies should be conducted to confirm the mechanisms *in vivo*. Macropinosomes in EGF-stimulated A431 cells have been shown to be difficult to fuse with lysosomes and to be recycled back to the extracellular fluid^24^,^57^,^58^, suggesting that digestion of exosomal contents might be prevented before the release of exosomal content from exosomes and macropinosomes in the cellular uptake pathway. The recycling is considered to affect the bioactivities of exosomal content inside cells, which are dependent on cellular mechanisms of macropinocytosis pathways.

Macropinocytosis has also been shown to play important roles for antigen presentation in dendritic cells and macrophages^59^,^60^, and the role of exosomes in their macropinocytotic cellular uptake has been hypothesised; further studies are urgently needed. Some pathogens, including *Salmonella typhimurium,* also induce macropinocytosis for their internalisation into the host cells^61^,^62^, and biological effects of exosomes in invasion and intracellular parasitism of the pathogen are expected. Thus, cell biological events based on macropinocytosis in physiological environments possibly include the cellular uptake of exosomes that contain biofunctional molecules, and further studies of the biological events from the view points of exosomes are required to elucidate their hidden mechanisms.

**Figure 3:**
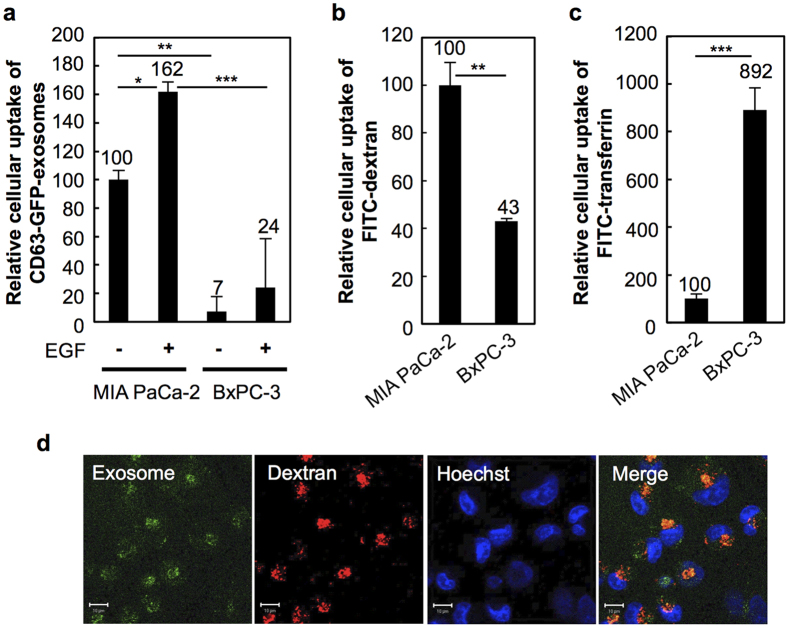
Macropinocytosis pathway by oncogenic Ras expression increases cellular uptake of exosomes. (**a**) Relative cellular uptake of CD63-GFP-exosomes (20 μg/ml) in MIA PaCa-2 or BxPC-3 cells in the presence or absence of EGF (500 nM) for 24 h at 37 °C, analysed using a flow cytometer. (**b**, **c**) Relative cellular uptake of FITC-dextran (**b**) or FITC-transferrin (**c**) in MIA PaCa-2 or BxPC-3 cells in the absence of EGF for 24 h at 37 °C, analysed using a flow cytometer. (**a**-**c**) The data are the averages (±SD) of three experiments. ^*^p < 0.05, ^**^p < 0.01, ^***^p < 0.001. (**d**) Confocal microscopic observation of MIA PaCa-2 cells treated with CD63-GFP-exosomes (20 μg/ml) in the presence of EGF (500 nM) in same experimental condition of (**a**). Green signals, CD63-GFP-exosomes; red signals, Texas Red-labelled dextran; blue signals, Hoechst 33342 for nuclear staining. Scale bar, 10 μm.

Stimulated macropinocytosis by expression of oncogenic Ras protein has been reported^48^,^49^,^50^. The *ras* family consists of three proto-oncogenes (H-*ras*, K-*ras*, N-*ras*), each of which acquires oncogenic properties by single mutations^48^. The mutated *ras* genes have been implicated in tumourigenesis^48^. Oncogenic K-Ras is a more potent Rac activator than H-Ras, leading to the induction of effective membrane ruffling^49^. Commisso *et al.* recently reported that Ras-transformed cells (MIA-PaCa-2 cells, homozygous K-Ras^G12C^ allele; NIH3T3 K-Ras^V12^ cells) use macropinocytosis as an important route for nutrient uptake in tumours^29^. Macropinocytotic cellular uptake of albumin was shown to sustain proliferation of oncogenic Ras-transformed cells by constituting a source of glutamine and other amino acids^29^. The growth efficacy of Ras-transformed pancreatic tumour xenografts was significantly reduced by the macropinocytosis inhibitor EIPA^29^. In our research, MIA PaCa-2 cells showed more effective macropinocytotic cellular uptake compared with wild-type K-Ras-expressing BxPC-3 cells (Fig. 3b), as previously reported^29^, and a high efficacy of cellular uptake of exosomes by MIA PaCa-2 cells was detected with further enhancement of cellular uptake by EGFR stimulation (Fig. 3a). On the other hand, iron is an essential nutrient for cancer cell proliferation, and iron (Fe(III))-loaded transferrin is transported into cells by binding to the transferrin receptor on the cell surface and the induction of clathrin-mediated transferrin endocytosis. The stimulation of the transferrin receptor by Fe(III)-loaded transferrin does not induce macropinocytosis. Interestingly, Fig. 3c shows a very high efficacy of transferrin uptake in BxPC-3 cells compared with that of MIA PaCa-2 cells. Thus, cellular biological methods for nutrient uptake are different in each tumour based on clathrin-mediated endocytosis and macropinocytosis, and macropinocytosis-mediated nutrient uptake might efficiently open routes for exosome uptake and the positive induction of cell regulation by exosomal contents.

**Figure 4:**
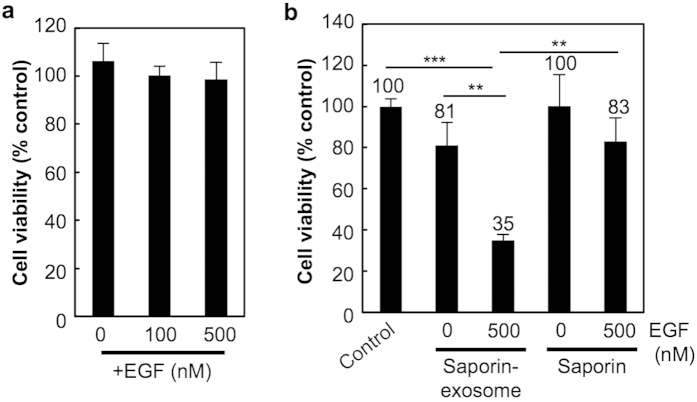
Saporin-encapsulated exosomes enhances cytotoxicity by EGF stimulation and macropinocytosis induction. (**a**) Cell viability of A431 cells treated with CD63-GFP-exosomes (20 μg/ml) in serum-free cell culture medium with or without co-treatment of EGF (100 or 500 nM) for 24 h at 37 °C, analysed using a WST-1 assay. (**b**) Cytotoxicity of saporin-encapsulated exosomes (4 μg/ml) or saporin (7 μg/ml) with or without co-treatment of EGF (500 nM). A431 cells were treated with each test sample in 10% FBS containing cell culture medium for 48 h at 37 °C, prior to a WST-1 assay. The data are the averages (±SD) of three experiments. ^**^p < 0.01, ^***^p < 0.001.

Using an exosome cellular uptake route by macropinocytosis, we successfully attained the delivery of ribosome-inactivating protein, saporin, by encapsulation in exosomes (Fig. 4b). As mentioned in the Results section, saporin inhibits protein synthesis in the cytosol, resulting in the growth inhibition of targeted cells^51^. Therefore, the effective cellular uptake of saporin-loaded exosomes and the cytosolic release of exosomal contents are needed in exosome-mediated intracellular delivery. The induction of macropinocytosis significantly enhanced the cytotoxicity of saporin-loaded exosomes, although a high concentration of saporin, which was not encapsulated in exosomes, showed slight effects for cytotoxicity even in the macropinocytosis induction (Fig. 4b). In the current experiments, the efficacy of cytosolic release of saporin from exosomes inside cells was not assessed, and the development of technology for the enhanced cytosolic release of exosomal contents is strongly needed for the effective delivery of therapeutic molecules using exosomes to attain their bioactivities in targeted cells as future works.

**Figure 5:**
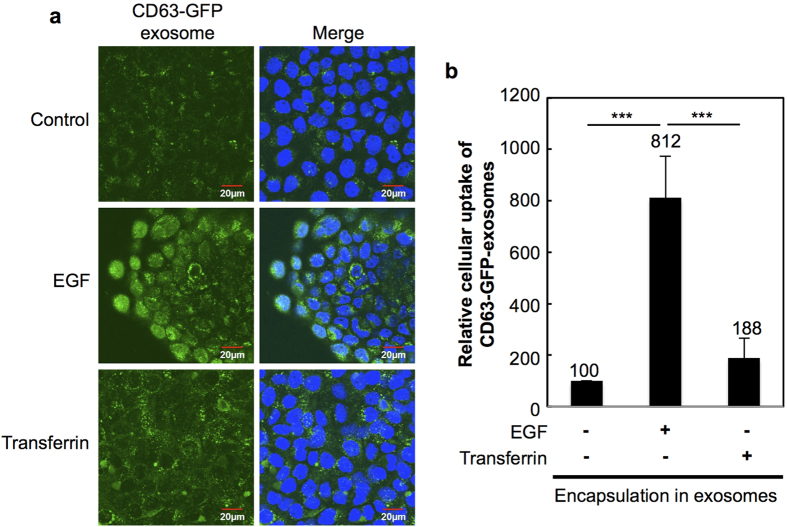
Encapsulation of EGF in exosomes enhances cellular uptake of exosomes. (**a**) Confocal microscopic observation of A431 cells treated with EGF- or transferrin-encapsulated CD63-GFP-exosomes (20 μg/ml) for 24 h at 37 °C. Green signals, CD63-GFP-exosomes; blue signals, Hoechst 33342 for nuclear staining. Scale bar, 20 μm. (**b**) Relative cellular uptake of CD63-GFP-exosomes with encapsulation of EGF or transferrin in the exosomes in the same experimental condition with (**a**), prior to analysis using a flow cytometer. The data represent the average (±SD) of three experiments. ^***^p < 0.001.

**Figure 6:**
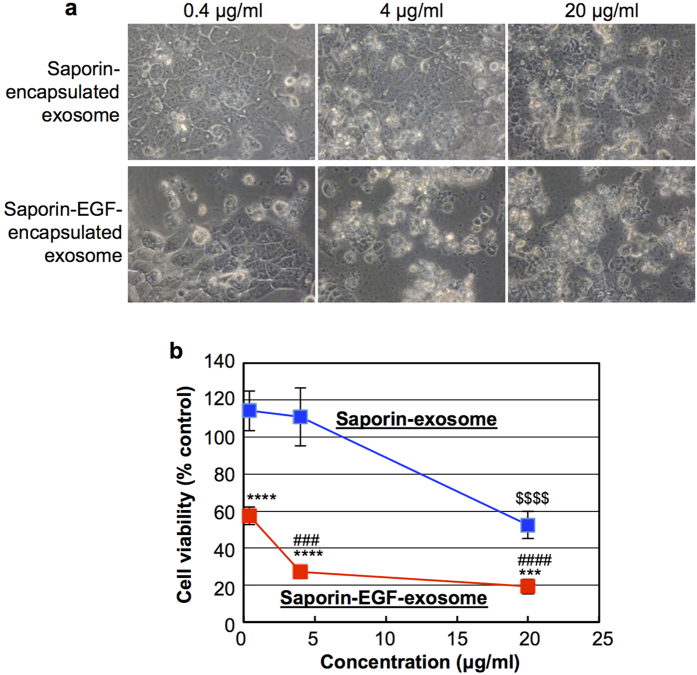
Effective cytotoxicity of saporin-EGF-encapsulated exosomes. (**a**) Microscopic observation of A431 cells treated with saporin- or saporin-EGF-encapsulated exosomes (0.4, 4, 20 μg/ml) for 72 h at 37 °C in 10% FBS-containing cell culture medium. (**b**) Cell viability of A431 cells treated with saporin- (blue line) or saporin-EGF- (red line) encapsulated exosomes (0.4, 4, 20 μg/ml) in the same experimental condition of (**a**) analysed using a WST-1 assay. The data represent the average (±SD) of four experiments. ^***^p < 0.001, ^****^p < 0.0001 versus saporin-encapsulated exosomes. ^$$$$^*p* < 0.0001 versus 0.4 μg/ml saporin-encapsulated exosomes. ^###^*p* < 0.001, ^####^*p* < 0.0001 versus 0.4 μg/ml saporin-EGF-encapsulated exosomes.

We also demonstrated that encapsulation of EGF in exosomes enhances their cellular uptake (Fig. 5). The delivery of growth factors using liposomes has been shown to be a useful technique for bone healing to stimulate mesenchymal cell migration and osteoblast differentiation^55^. Considering the characteristics of EGF recycling through endocytosis and exocytosis^56^, the release of EGF from exosomes in the endocytosis pathway possibly induces EGF recycling, and secreted EGF from the cells by exocytosis stimulates EGFR in plasma membranes. As mentioned, macropinosomes in EGF-stimulated A431 cells have been shown to be recycled back to the extracellular fluid^24^,^57^,^58^. This method only relies on EGF encapsulation in exosomes without any modifications using exosome membrane proteins or lipids. Because covalent modification (e.g., conjugation with chemical linkers, gene expression engineering) of membrane proteins and lipids possibly affect their biological functions, this simple technique of EGF encapsulation in exosomes is considered a beneficial method for maintaining the exosome membrane environments, although detailed studies are needed to assess the release and recycling of EGF, which is encapsulated in exosomes during their cellular uptake. In addition, co-encapsulation of EGF and saporin showed significant induction of cytotoxicity (Fig. 6). This simple methodology will be able to be applied to a wide variety of therapeutic molecules in exosome-mediated intracellular delivery for controlling cellular functions.

In conclusion, we have demonstrated important roles of macropinocytosis in the efficient cellular uptake of exosomes. The macropinocytosis pathway, which is induced by cellular biological and environmental conditions, including related-receptor activation, oncogenic protein expression, and pathogen invasion, is possibly associated with the cellular uptake of exosomes leading to cellular regulation and cell-to-cell communications. Although further studies on macropinocytosis-dependent cellular uptake of exosomes are needed, our findings will contribute to biological, pharmaceutical, and medical research fields for the understanding of biological functionalities of exosomes in various cellular events and conditions (e.g., cell-to-cell communications, cancer and other diseases, and infection) under stimulated macropinocytosis and for the development of exosome-mediated delivery systems for future therapeutic and diagnostic applications.

## Methods

### Cell culture

Human cervical cancer-derived HeLa cells, human pancreas carcinoma-derived MIA PaCa-2 cells, and human pancreas adenocarcinoma-derived BxPC-3 cells were purchased from the Riken BRC Cell Bank (Ibaraki, Japan). The human epidermoid carcinoma-derived A431 cells were purchased from the American Type Culture Collection (Manassas, VA, USA). The cells were cultured in minimum essential medium α (α-MEM; Gibco, Life Technologies Corporation, Grand Island, NY, USA) containing 10% heat-inactivated foetal bovine serum (FBS; Gibco, Life Technologies Corporation) (HeLa cells), minimum essential medium (MEM) (Gibco, Life Technologies Corporation) containing 10% heat-inactivated FBS (Gibco, Life Technologies Corporation) (A431 cells), Eagle’s minimum essential medium (EMEM) (Wako, Osaka, Japan) containing 10% FBS (HyClone Laboratories, South Logan, UT, USA), 0.1 mM MEM non-essential amino acids (Gibco), penicillin-streptomycin (50 units/ml and 50 μg/ml) (Sigma-Aldrich, St. Louis, MO, USA) (MIA PaCa-2 cells), and RPMI1640 (Gibco, Life Technologies Corporation) containing 10% FBS (HyClone), and penicillin-streptomycin (50 units/ml and 50 μg/ml) (Sigma-Aldrich) (BxPC-3 cells). Cells were grown on 100-mm dishes and incubated at 37 °C under 5% CO_2_.

### Preparation of HeLa cells stably expressing green fluorescent protein (GFP)-fused CD63

Tetraspanin CD63 is a membrane marker protein of exosomes. We prepared HeLa cells stably expressing GFP-fused CD63 to secrete CD63-GFP-containing exosomes (CD63-GFP-exosomes). HeLa cells (4.7 × 10^4^ cells) were plated on a 24-well microplate (Iwaki, Tokyo, Japan) and incubated for 1 day. The cells were transfected with CD63-GFP plasmid (pCT-CD63-GFP, pCMV, Cyto-Tracer, System Biosciences, Mountain View, CA, USA; 800 ng) complexed with Lipofectamine LTX reagent (2 μl) with PLUS reagent (1 μl; Invitrogen, Life Technologies Corporation) in α-MEM containing 10% FBS (200 μl). The cells were also treated with puromycin (3 μg/ml; LKT Laboratories, St. Paul, MN, USA) for the antibiotic selection of HeLa cells stably expressing CD63-GFP (CD63-GFP-HeLa).

### Isolation of exosomes

CD63-GFP-HeLa cells (2 × 10^6^ cells) were seeded on 100-mm dishes in α-MEM (10 ml) containing 10% FBS and puromycin (3 μg/ml) and incubated for 1 day at 37 °C under 5% CO_2_. The cells were washed with serum-free α-MEM (five times, 5 ml) and incubated in α-MEM (10 ml) containing 10% exosome-free FBS (EXO-FBS, ATLAS biological, Fort Collins, CO, USA) and puromycin (3 μg/ml; LKT Laboratories) for 3 days. The cell culture medium was collected, and secreted exosomes were isolated using Total Exosome Isolation (from cell culture media) (Invitrogen, Austin, TX, USA). The concentrations of the isolated exosomes are described in terms of their protein concentrations, which were determined using a Pierce BCA protein assay kit (Thermo Fisher Scientific Inc., Rockford, IL, USA).

Exosome isolation was conducted using ultracentrifugation^63^. The collected cell culture medium was centrifuged (300 × *g*) for 10 min at 4 °C. The supernatant was centrifuged (2,000 × *g*) for 10 min at 4 °C and again (10,000 × *g*) for 30 min at 4 °C to remove cell debris. The supernatant was then centrifuged (100,000 × *g*) for 70 min at 4 °C using an ultracentrifuge (Himac CP65β, Hitachi, Tokyo, Japan) in duplicate, and the pellet was collected in Dulbecco’s phosphate buffered saline (PBS; Nacalai Tesque, Kyoto, Japan).

### Preparation of HeLa cells expressing epidermal growth factor receptor (EGFR)

HeLa cells (2 × 10^5^ cells) were plated on a 35-mm glass-based dish (Iwaki, Tokyo, Japan) and incubated in α-MEM containing 10% FBS for 24 h at 37 °C under 5% CO_2_. The cells were transfected with an EGFR plasmid (800 ng)^64^ complexed with Lipofectamine LTX reagent (2 μl) with PLUS reagent (1 μl; Invitrogen, Life Technologies Corporation) in α-MEM containing 10% FBS (200 μl) for 24 h at 37 °C under 5% CO_2_.

### Electron microscopy

Suspended exosomes in PBS (30 μg/ml) were dropped on a carbon-coated grid (400 mesh) and washed with distilled water. Uranil acetate was applied to the grid and left for 10 sec at room temperature. Then, the reagent was removed with filter paper and dried, prior to imaging with a transmission electron microscope (TEM; JEM1200EX, JEOL, Tokyo, Japan).

### Confocal microscopy

Cells (2 × 10^5^ cells, 2 ml) were plated on 35-mm glass-based dishes (Iwaki) and incubated for 24 h at 37 °C under 5% CO_2_. After complete adhesion, the cells were washed with cell culture medium (three times, 1 ml) and treated with exosome samples in the presence or absence of human EGF (Sigma-Aldrich; 200 μl/well). The cells were stained with Hoechst 33342 (Invitrogen; 5 μg/ml) for 15 min at 37 °C, prior to cell washing. The cells were then washed with fresh cell culture medium (three times, 1 ml) and analysed using a FV1200 confocal laser scanning microscope (Olympus, Tokyo, Japan) equipped with a 40x objective without cell fixation. For the detection of macropinocytosis uptake, co-treatment with Texas Red-dextran (70 kDa, 0.5 mg/ml; Molecular Probes, Eugene, OR, USA) with exosome samples was conducted.

### Flow cytometry

Cells (4.7 × 10^4^ cells, 1 ml) were plated on a 24-well microplate (Iwaki) and incubated for 24 h at 37 °C under 5% CO_2_. After complete adhesion, the cells were washed with cell culture medium (three times, 200 μl) and treated with exosome samples, FITC-dextran (70 kDa, 0.5 mg/ml; Sigma-Aldrich), or FITC-transferrin (0.5 μg/ml; Rockland, Gilbertsville, PA, USA) (each 200 μl/well), prior to washing with PBS (three times, 200 μl). The cells were then treated with trypsin (0.1 g/l)-ethylenediaminetetraacetic acid (EDTA; 0.11 mmol/l; Nacalai Tesque; 200 μl/well) at 37 °C for 10 min, prior to the addition of PBS (200 μl), and centrifuged at 3,000 rpm (800 × *g*) for 3 min at 4 °C. After removal of the supernatant, the cells were washed with PBS (400 μl) and centrifuged at 3,000 rpm for 3 min at 4 °C. This washing cycle was repeated, and the cells were suspended in PBS (400 μl) and subjected to fluorescence analysis on a Guava EasyCyte (Merck Millipore, Billerica, MA, USA) flow cytometer using 488 nm laser excitation and a 525 nm emission filter. Living cells (10,000 cells/sample) for the detection of cellular fluorescence intensity were counted based on forward scatter and side scatter analyses. For experiments using the macropinocytosis inhibitor, 5-(*N*-ethyl-*N*-isopropyl)amiloride (EIPA; Sigma-Aldrich), cells were pre-treated with EIPA (20 μM) for 30 min at 37 °C before treatment with exosome samples in EIPA (20 μM)-containing cell culture medium (200 μl/well).

### Preparation of FITC-labeled saporin and FITC-labeled EGF

Saporin (200 μg, saporin from *Saponaria officinalis* seeds, Sigma-Aldrich) or EGF (200 μg) dissolved in H_2_O (100 μl) was mixed with FITC (2 equivalents, Sigma-Aldrich) dissolved in dimethyl sulfoxide (10 μl) and *N,N*-diisopropylethylamine (0.5 μl) at 30 °C for 2 h. Gel filtration on a Sephadex G-25 column (PD-10, GE Healthcare) was conducted for the removal of unreacted FITC prior to lyophilisation. Concentration of the protein was determined using a Pierce BCA protein assay kit (Thermo Fisher Scientific Inc.).

### Encapsulation of proteins into exosomes

To load saporin into exosomes (saporin from *Saponaria officinalis* seeds, Sigma-Aldrich), CD63-GFP-exosomes (25 μg) were mixed with saporin (50 μg) in PBS (100 μl). After electroporation (poring pulse: twice pulse (5 msec), transfer pulse: five pulse (20 V, 50 msec)) in a 1-cm electroporation cuvette at room temperature using a super electropolater NEPA21 TypeII (NEPA genes, Tokyo, Japan), the removal of unencapsulated proteins was accomplished by washing and filtration using Amicon Ultra centrifugal filters (100 K device, Merck Millipore; triple washing with 500 μl PBS, 18,000 × *g*, 4 °C, 10 min). For encapsulation of saporin with human EGF or human transferrin (Sigma-Aldrich), EGF (25 μg) or transferrin (100 μg) was added to saporin and CD63-GFP-exosome samples before electroporation.

FITC-labeled saporin loaded in exosomes were confirmed using a spectrofluometer (FP-6200, JASCO, Tokyo, Japan). The electroporation method was optimized for poring pulse (50, 100, 200, 300 V), resulting in encapsulation of FITC-labeled saporin (45, 65, 120, 105 ng/ml) in 20 μg/ml of exosomes, respectively. Therefore, we used an experimental poring pulse of 200 V in the electroporation steps. The efficiency of saporin encapsulation into exosomes was calculated to be 0.2%. In the same electroporation condition (200 V), the concentration of EGF or transferrin encapsulated in 20 μg/ml exosomes was estimated to be around 550 ng/ml (EGF) or 1.8 μg/ml (transferrin) using the FITC-labeled EGF and FITC-labeled transferrin (Molecular Probes). The efficiency of encapsulation into exosomes was calculated to be 2.8% (EGF) and 2.3% (transferrin).

### Cell viability (WST-1 assay)

Cell viability was analysed using a 4-[3-(4-iodophenyl)-2-(4-nitrophenyl)-2H-5-tetrazolio]-1,3-benzene disulfonate (WST-1) assay as previously described^65^. Cells (1 × 10^4^ cells, 100 μl) were incubated in 96-well microplates for 24 h at 37 °C under 5% CO_2_. The cells were then treated with exosome samples (50 μl) for each experimental period at 37 °C under 5% CO_2_. After the sample treatment, WST-1 reagent (10 μl) was added to each well, and the samples were incubated for 45 min at 37 °C. Absorbance was measured at 450 nm (A_450_) and 620 nm (A_620_), and the value obtained by subtracting A_620_ from A_450_ corresponded to the viable cell number.

### Western blot analysis

Isolated exosomes were added to lysis buffer (62.5 mM Tris-HCl (pH = 6.8), 2% SDS, 10% glycerol, 0.002% bromophenol blue, 5% 2-mercaptoethanol). The boiled samples were separated by 10% SDS-PAGE, transferred to polyvinylidene fluoride (PVDF) membranes (GE Healthcare, Pittsburgh, PA, USA), and treated with anti-CD63 antibody (TS63, Abcam, Cambridge, UK). A secondary antibody labelled with horseradish peroxidase (anti-mouse IgG HRP-linked whole antibody, GE Healthcare) was used, and immunoreactive species were detected by an Enhanced Chemiluminescence (ECL) Plus Western Blotting Detection System (GE Healthcare). For the detection of EGFR, A431 cells (2 × 10^5^ cells, 2 ml) were plated on a 12-well microplate (Iwaki) and incubated for 24 h at 37 °C under 5% CO_2_. After complete adhesion, the cells were washed with cell culture medium (triple washing, 200 μl) and treated with EGF (100 nM) for 1 min at 37 °C. After EGF treatment, the cells were scraped in lysis buffer (200 μl). The boiled lysate samples were separated by SDS-PAGE and transferred to PVDF membranes as described above. The membranes were treated with a p-EGFR (Tyr1173) antibody (sc-12351, Santa Cruz Biotechnology), and an EGFR antibody (sc-03, Santa Cruz Biotechnology), and a secondary antibody labelled with horseradish peroxidase (anti-rabbit IgG HRP-linked whole antibody, GE Healthcare) was used, prior to the detection of immunoreactive species.

### Statistic analyses

All statistical analyses were performed using GraphPad Prism software (ver. 5.00; GraphPad, San Diego, CA, USA). For comparison of two groups, unpaired Student’s t-test was used after verification of the equal variances by F-test. The Welch’s correction was performed when the variances across groups are assumed to be unequal. For multiple comparison analyses, either one-way analysis of variance (ANOVA) followed by Tukey’s post-hoc test or two-way ANOVA followed by Bonferroni’s post-hoc test were used. Differences were considered significant when the calculated *p* - value was <0.05.

## Additional Information

**How to cite this article**: Nakase, I. *et al.* Active macropinocytosis induction by stimulation of epidermal growth factor receptor and oncogenic Ras expression potentiates cellular uptake efficacy of exosomes. *Sci. Rep.* **5**, 10300; doi: 10.1038/srep10300 (2015).

## Supplementary Material

Supplementary Information
