# Supplementary material for: Active macropinocytosis induction by stimulation of epidermal growth factor receptor and oncogenic Ras expression potentiates cellular uptake efficacy of exosomes
Source: Sci Rep. 2015 Jun 3;5:10300. doi: 10.1038/srep10300 (PMC4453128; doi:10.1038/srep10300)
Supplement: Supplementary Information [file srep10300-s1.pdf]

Scientific Reports

Supplementary information

Title:

Active macropinocytosis induction by stimulation of epidermal growth factor receptor and oncogenic Ras expression potentiates cellular uptake efficacy of exosomes

Authors:

Ikuhiko Nakase[1,*], Nahoko Bailey Kobayashi[2,3], Tomoka Takatani-Nakase[4], Tetsuhiko Yoshida[2,3,*]

Affiliations:

[1]Nanoscience and Nanotechnology Research Center, Research Organization for the 21st Century, Osaka Prefecture University, Naka-ku, Sakai, Osaka 599-8570, Japan

[2]Keio Advanced Research Centers (KARC), Keio University, Tsukuba, Ibaraki 300-2611, Japan

[3]Institute for Advanced Sciences, Toagosei Co., Ltd., Tsukuba, Ibaraki 300-2611, Japan

[4]Department of Pharmaceutics, School of Pharmacy and Pharmaceutical Sciences, Mukogawa Women's University, 11-68, Koshien Kyuban-cho, Nishinomiya, Hyogo 663-8179, Japan

*Correspondence and requests for materials should be addressed to I.N. (email: i-nakase@21c.osakafu-u.ac.jp) or T.Y. (email: tyoshida@dmb.med.keio.ac.jp).

Figure captions

Supplementary Figure 1. **Secretion of CD63-GFP-exosomes from HeLa cells.** (**a**) Confocal microscopic observation of CD63-GFP-HeLa cells. Scale bar, 20 μm. (**b**) TEM observation of CD63-GFP-exosomes. Scale bar, 100 nm. (**c**) Western blot showing exosomes secreted from HeLa cells. The CD63 exosome marker protein was detected as described in the Materials section.

Supplementary Figure 2. **Induction of macropinocytosis by stimulation of EGFR with EGF.** (**a**) Western blot of phosphorylation of EGFR Y1173 (EGFR pY1173) of A431 cells stimulated with EGF (100 nM) for 1 min at 37 °C. (**b**) Morphological changes of A431 cells treated with EGF (100 nM) for 10 min at 37 °C. The arrows indicate representative membrane rufflings induced by EGF. (**c**) Confocal microscopic observation of A431 cells treated with Texas Red-dextran (70 kDa, 0.5 mg/ml) containing cell culture medium in the presence or absence of EGF (500 nM) for 24 h at 37 °C. Red signals, Texas Red-dextran; blue signals, Hoechst 33342 for nuclear staining. Scale bar, 20 μm. (**d**) Relative cellular uptake of FITC-dextran in same experimental condition of (**c**) analysed using a flow cytometer. The data are the averages (± SD) of three experiments. $^{**}p < 0.01$.

Supplementary Figure 3. **Stimulation of EGFR by continuous treatment of EGF enhances cellular uptake of exosomes.** (**a**) Confocal microscopic observation of A431 cells treated with CD63-GFP-exosomes (20 μg/ml) in the presence or absence of EGF (500 nM)/day for 96 h at 37 °C. Green signals, CD63-GFP-exosomes; blue signals, Hoechst 33342 for nuclear staining. Scale bar, 20 μm. (**b**) Relative cellular uptake of CD63-GFP-exosomes in same experimental condition of (**a**) analysed using a flow cytometer. The data are the averages (± SD) of three experiments. $^{***}p < 0.001$.

Supplementary Figure 4. **Increased EGFR expression enhances internalisation of exosomes by cells.** Confocal microscopic observation of wild-type (WT) or EGFR-highly expressing HeLa cells treated with CD63-GFP-exosomes (20 μg/ml) in the presence of EGF (500 nM) at 37 °C. Green signals, CD63-GFP-exosomes; blue signals, Hoechst 33342 for nuclear staining. Scale bar, 20 μm.

Supplementary Figure 5. **High voltage of electroporation affects aggregation of exosomes.** (**a**) TEM observation of CD63-GFP-exosomes after electroporation (poring pulse: twice pulse (200 V, 5 msec), transfer pulse: five pulse (20 V, 50 msec)). Scale bar, 100 nm. (**b**) Confocal microscopic observation of FITC-saporin-encapsulated exosomes (500 ng/ml) (without CD63-GFP expression) after electroporation (poring pulse: twice pulse (0, 200, or 300 V)). Arrows show typical aggregation of FITC-saporin-encapsulated exosomes. Scale bar, 50 μm.

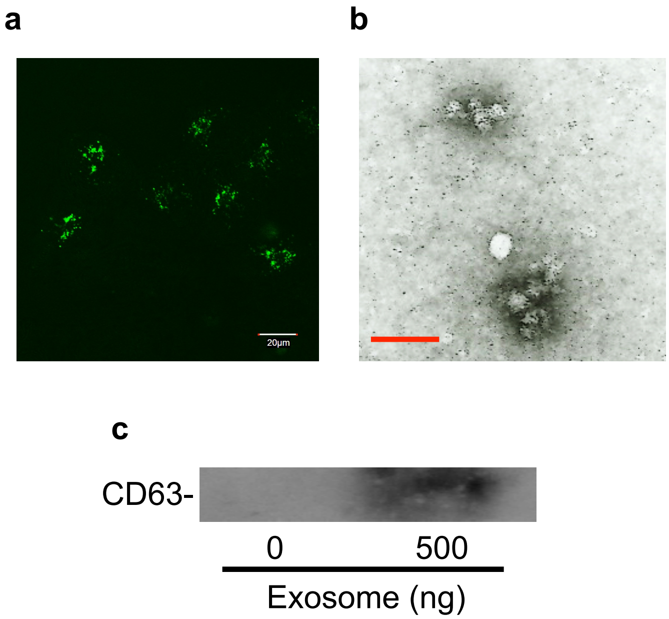

Supplementary Figure 1

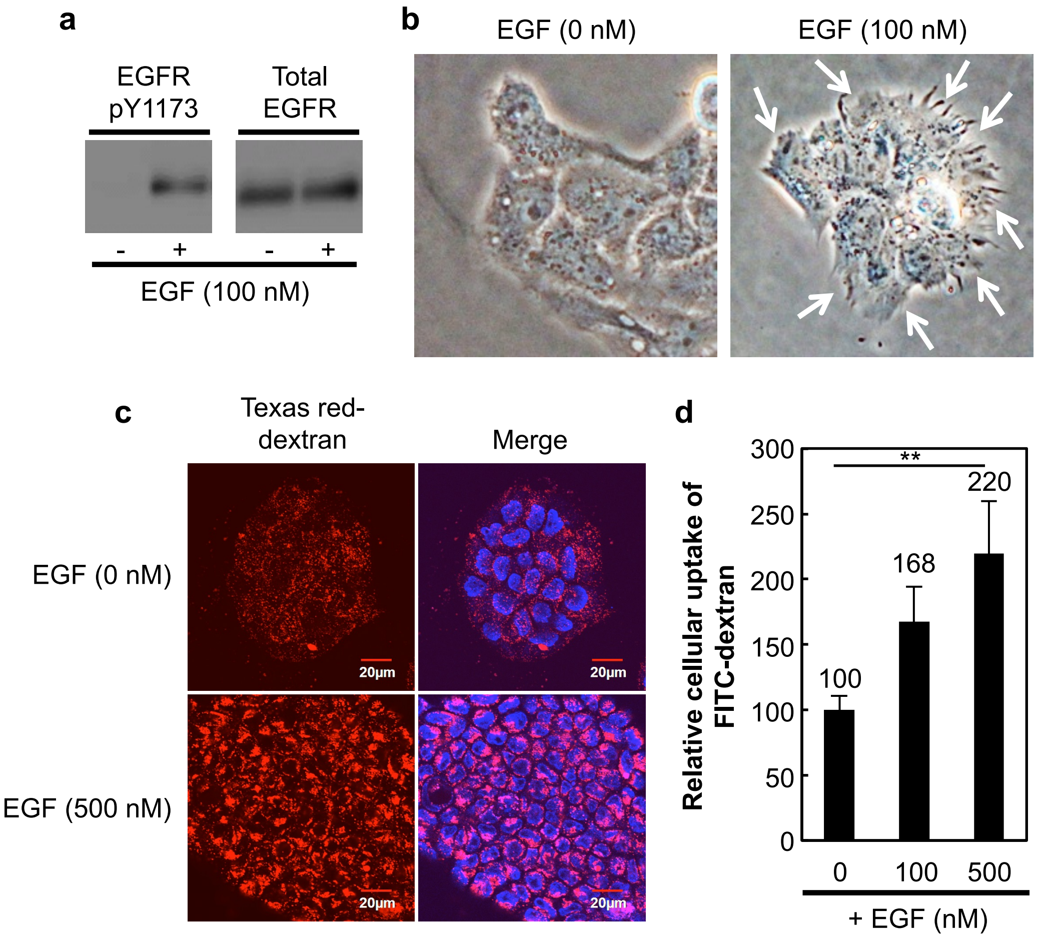

Supplementary Figure 2

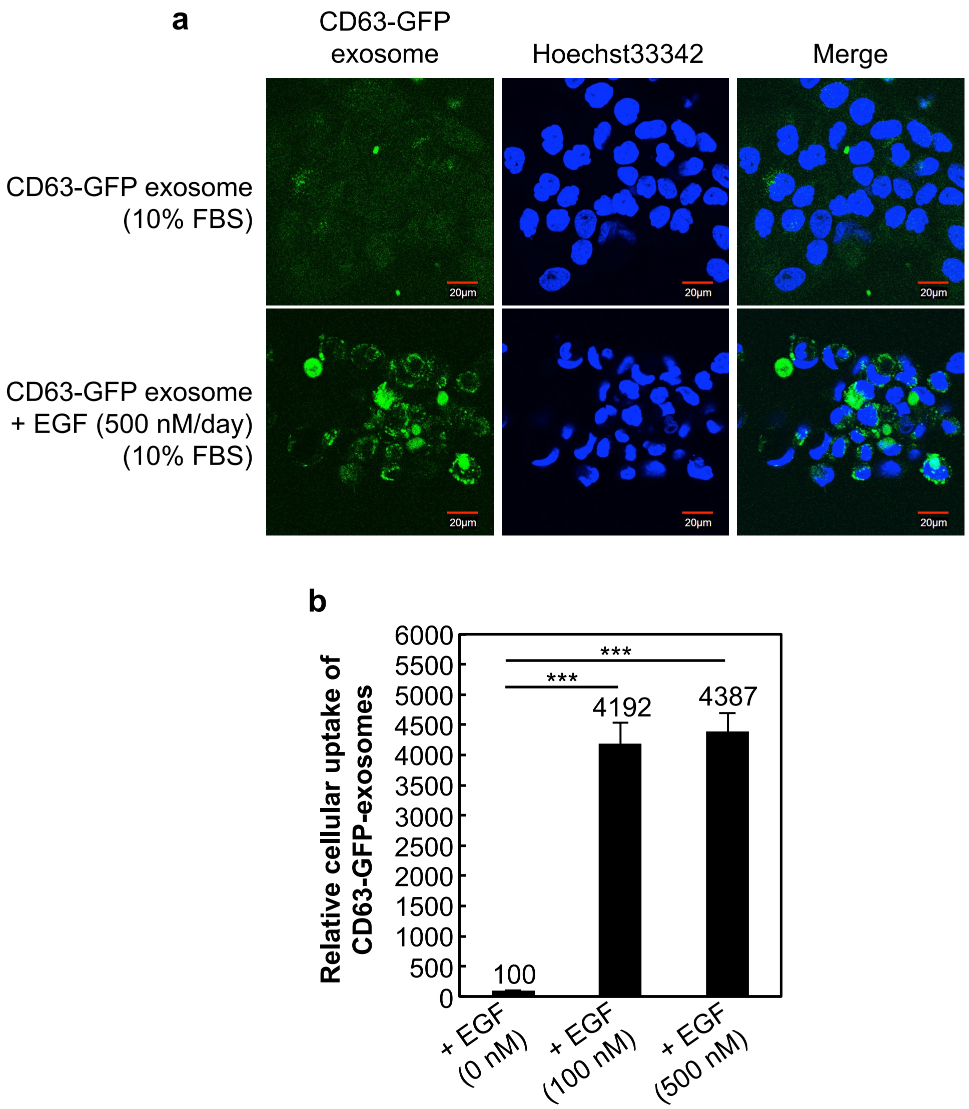

Supplementary Figure 3

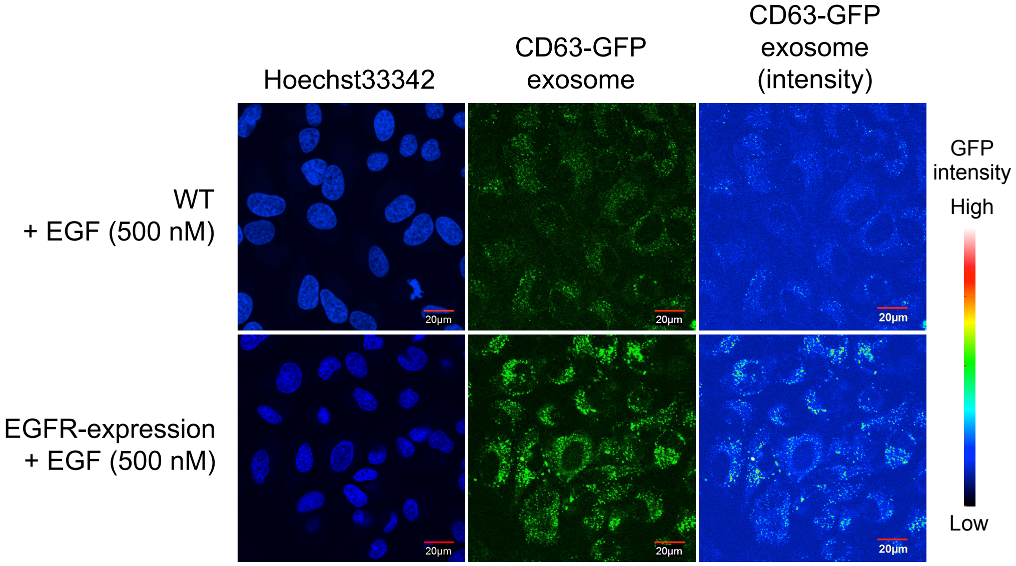

Supplementary Figure 4

a

b

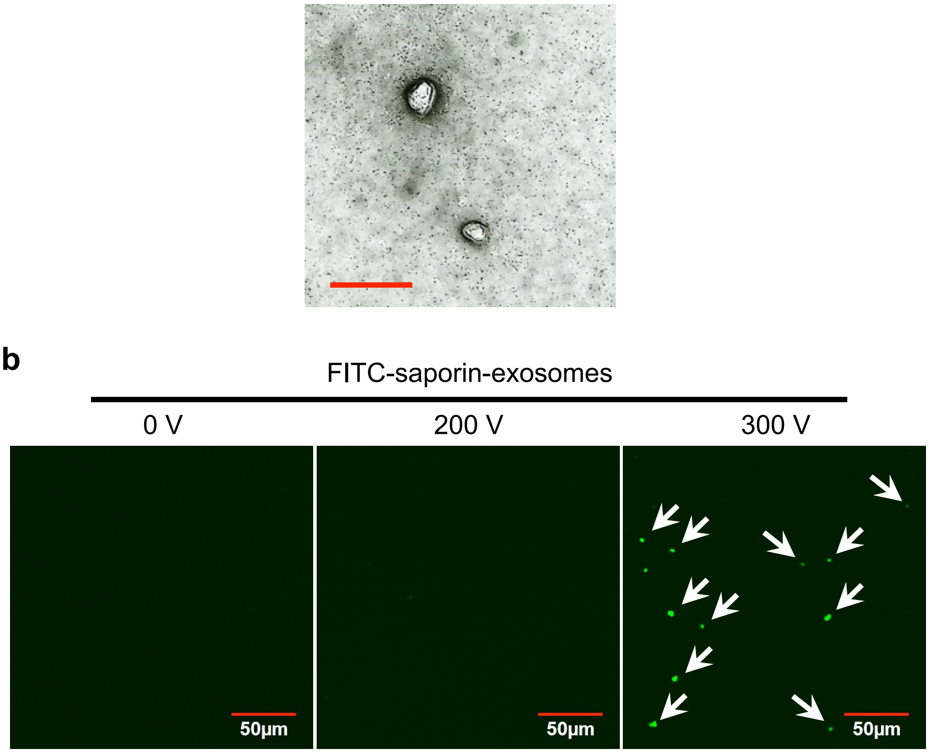

Supplementary Figure 5
